# Axon Myelin Transfer of a Non-Enveloped Virus

**DOI:** 10.1371/journal.pone.0001331

**Published:** 2007-12-26

**Authors:** Jean-Pierre Roussarie, Claude Ruffié, Julia M. Edgar, Ian Griffiths, Michel Brahic

**Affiliations:** 1 Department of Virology, URA3015 Centre National de la Recherche Scientifique (CNRS), Institut Pasteur, Paris, France; 2 Applied Neurobiology Group, Institute of Comparative Medicine, University of Glasgow, Glasgow, United Kingdom; Institut Pasteur, France

## Abstract

We showed previously that Theiler's virus, a neurotropic non-enveloped picornavirus of mouse, traffics from the axon of infected neurons into the surrounding myelin. When this traffic is interrupted, as in the *shiverer* mouse which bears a mutation in the myelin basic protein gene, the virus is unable to persist in the central nervous system. In the present work, we used the *Wld^s^* mutant mouse, a strain in which axonal degeneration is considerably slowed down, to show that axon to myelin traffic takes place in the absence of axon degeneration. Our results suggest the existence of a mechanism of transfer of axonal cytoplasm into the myelin which Theiler's virus might exploit to ensure its persistence.

## Introduction

Myelin is an extension of the oligodendrocyte cell body wrapped many times around the axon. Myelin is important not only for rapid saltatory conduction of nerve impulse but also for support of the underlying axon [1]. For example, inactivating the *Cnp1* myelin gene does not alter myelin ultrastructure but causes accumulation of membranous organelles in the axons, axon swelling and degeneration [2]. This support role, and the exact way by which oligodendrocytes and axons communicate, is still poorly understood. Theiler's virus (TMEV) is a mouse picornavirus responsible for a persistent infection of CNS and a chronic, inflammatory, neurological disease [3]. TMEV infects neurons first but persists in oligodendrocytes and macrophages in the white matter. In a previous paper we showed that TMEV infects retinal ganglion neurons when injected in the eye, is transported axonally in the optic nerve, and infects optic nerve oligodendrocytes through their myelin sheaths [4]. We also showed that a deletion of the myelin basic protein gene prevents this axon-myelin traffic and renders the mice resistant to persistent infection [4], [5]. This observation raises important questions regarding the mechanism of axon to myelin traffic and the role of this traffic in viral persistence. Picornaviruses, such as TMEV, are non-enveloped and cannot travel from cell to cell by budding followed by fusion. Classically, they exit infected cells by lysis, although exit without lysis has been discussed but not formally proven [6]. In the present work we investigated the role of axon lysis in the entry of TMEV into the myelin using the *Wld^s^* mouse, a mutant strain in which axonal degeneration is 10 times slower than in wild type controls [7]. We show that TMEV enters the myelin even when the axons do not degenerate.

## Results

### The *Wld^s^* mutation does not affect the infection of retina by TMEV or the axonal transport of the virus in the optic nerve

**Figure 1:**
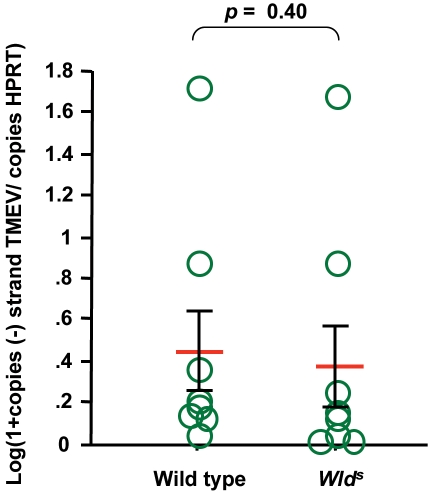
Amount of viral RNA in the retina, 2 days post-inoculation. Total RNA was extracted from the eye. The number of TMEV negative-strand RNA copies was measured in each sample by real time RT-PCR. Negative-strand RNA is diagnostic of replication whereas positive-strand RNA can be residual inoculum. Each circle corresponds to a different mouse. There is no statistically significant difference of viral load between control and *Wld^s^* mice (*p* = 0.40, Mann-Whitney test).

**Figure 2:**
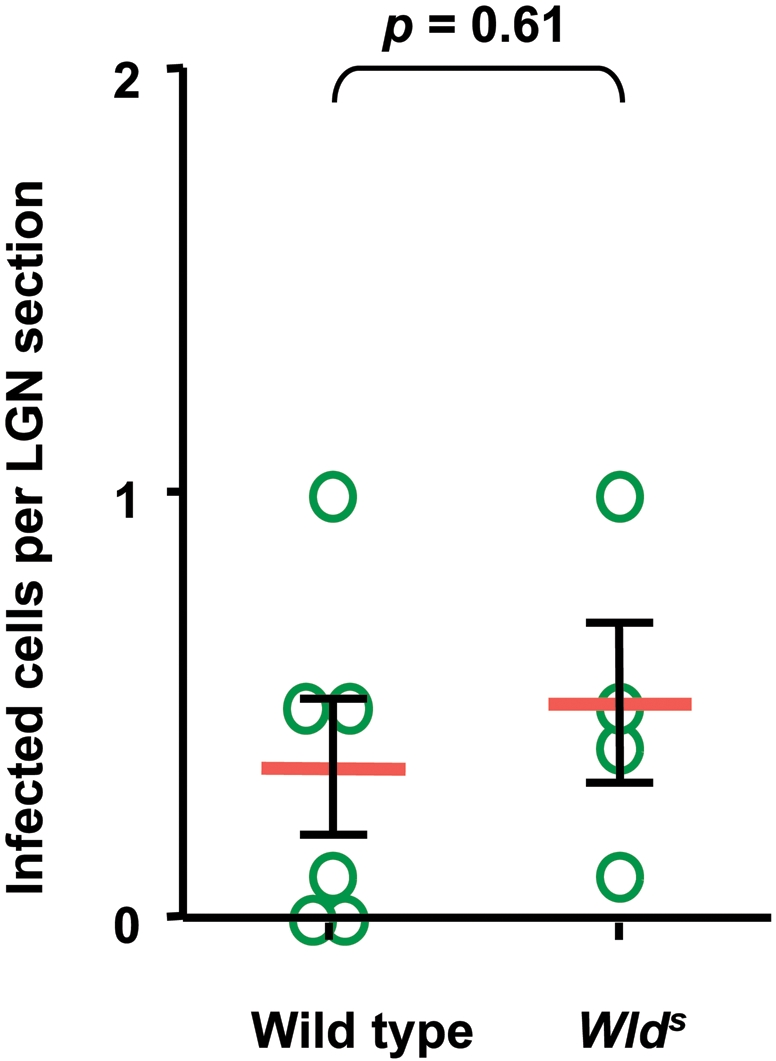
Number of infected cells in the LGN, 4 days post-inoculation. Mice were inoculated intraocularly in one eye. Serial sections of the entire corresponding geniculate nucleus were stained with a fluorescent anti-viral capsid antibody. The total number of fluorescent neuron cell body was counted. Each circle corresponds to a different mouse. There is no statistically significant difference of viral load between control and *Wld^s^* mice (*p = *0.61, Mann-Whitney test).

**Figure 3:**
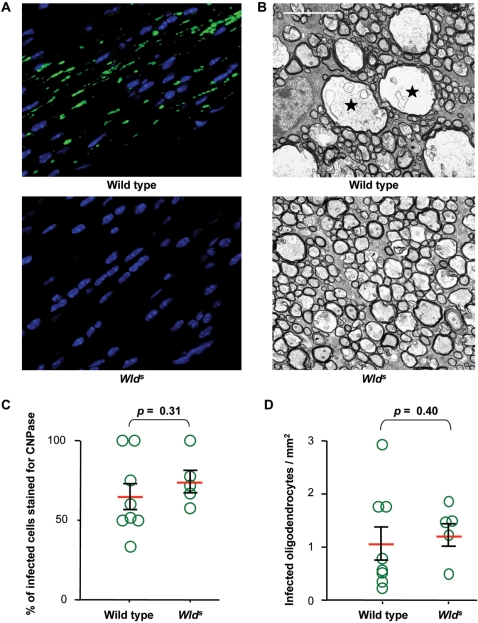
Phenotype of TMEV-infected optic nerves of wild type and *Wld^s^* mice. A: Longitudinal sections of optic nerves reacted with the SMI32 antibody (green). Nuclei were stained with DAPI (blue). SMI32 staining was detected in wild type samples only. B: Electron microscopy. Swollen axons with disorganized cytoskeleton are present in wild type mice only (star); bar = 5 µm. Mice shown in A and B were sacrificed 3 days post inoculation. C: Frozen sections were stained for CNPase (oligodendrocytes) or GFAP (astrocytes) and for TMEV to determine the percentage of TMEV^+^ cells which were CNPase^+^. No difference between wild type and *Wld^s^* mice was found (*p* = 0.31, Mann-Whitney test). D: The same sections were used to measure the density of CNPase^+^, TMEV^+^ cells (infected oligodendrocytes/mm^2^). No difference between wild type and *Wld^s^* mice was found (*p* = 0.40, Mann-Whitney test). In C and D, each circle corresponds to a different mouse.

Wild type and *Wld^s^* mice were inoculated with TMEV in the vitreous chamber of the eye as described [4]. First, we examined if the *Wld^s^* mutation altered the infection of retinal ganglion neurons. The amount of viral negative-strand RNA in retina was measured by real-time RT-PCR 2 days post-inoculation. Negative-strand RNA is diagnostic of viral replication, as opposed to genomic positive-strand RNA which can come from the inoculum as well as from newly replicated genomes. No difference was observed between the 2 strains of mice (Figure 1). Next we examined the effect of the *Wld^s^* mutation on the axonal transport of the virus by looking for viral antigens in neurons of the lateral geniculate nucleus (LGN), to which retinal ganglion cells project, 4 days post-inoculation. Serial sections of the entire nucleus were stained with fluorescent antibodies specific for TMEV capsid antigens and the total number of positive cell bodies was counted. As shown in Figure 2, no statistically significant difference was found between wild type and *Wld^s^* mice. This result suggested that the mutation did not affect the rate of anterograde axonal transport of the virus. We then studied the effect of infection on axonal degeneration using both immunofluorescence with the SMI32 monoclonal antibody, an antibody which stains non-phosphorylated neurofilaments specifically, and electron microscopy. Wild type and *Wld^s^* optic nerves were obtained 3 and 4 days post-inoculation, coded and observed blindly. In the wild type mice, both at 3 and 4 days post-inoculation, immunofluorescence showed conspicuous staining with the SMI32 antibody and electron microscopy showed numerous swollen axons with disorganized cytoskeleton. In contrast, in *Wld^s^* mice, no SMI32 staining was observed by immunofluorescence and abnormal axons were observed only very occasionally by electron microscopy (Figure 3A, B). Thus, the *Wld^s^* mutation did not affect the infection of the retina by TMEV or the axonal transport of the virus in the optic nerve but prevented axonal degeneration.

### The axon to myelin traffic of TMEV takes place in the absence of axon degeneration

We then asked if oligodendrocytes in the optic nerves of *Wld^s^* mice were infected 4 days post-inoculation, the earliest time at which infected glial cells are detected in this nerve in wild type mice. An early time was chosen to rule out the possibility of glia to glia spread of the infection. The mice were inoculated in one eye only in order to use the contralateral pre-chiasmatic segment of the optic nerve as control for the hematogenous spread of virus from axon to myelin. Frozen sections were coded, doubly-stained with fluorescent antibodies against 2′–3′cyclic nucleotide phosphodiesterase (CNPase), an oligodendrocyte marker, or glial fibrillary acidic protein antibodies (GFAP), an astrocyte marker, and with fluorescent anti-TMEV antibodies. Optical sections obtained with the Apotome fluorescent microscope were scanned blindly, and doubly-labeled cells were quantified as described [4]. No virus was found in the contralateral pre-chiasmatic segment, ruling out hematogenous spread. In contrast, infected cells were present in the ipsilateral nerve in both wild type and *Wld^s^* mice. The percentage of infected cells that were CNPase^+^ was the same for wild type and *Wld^s^* mice (65%±8% and 75%±7% respectively; *p* = 0.31, Mann-Whitney test) (Figure 3C). The CNPase^−^ infected cells were GFAP^+^ astrocytes. The number of infected oligodendrocytes per mm^2 ^of optic nerve section was also the same for both mice (1.1±0.3 and 1.3±0.2 oligodendrocytes/mm^2^; *p* = 0.40, Mann-Whitney test) (Figure 3D). Therefore the *Wld^s^* mutation did not prevent the infection of optic nerve oligodendrocytes from the axons of retinal ganglion neurons.

## Discussion

Our results show that the *Wld^s^* mutation does not alter the extent of replication of TMEV in retina or the axonal transport of the virus from retinal ganglion cells to neurons of the LGN. They show a dramatic difference in axonal degeneration between the optic nerves of intra-ocularly inoculated wild type and *Wld^s^* mice. In spite of this difference, Figure 3 shows that the extent of oligodendrocyte infection was the same in both types of mice. The absence of infection in the contralateral optic nerve ruled out hematogenous spread. It is not possible to formally exclude the presence of limited axonal degeneration, undetected by SMI32 staining, in *Wld^s^* mice. However, if axonal degeneration was the cause of oligodendrocyte infection in wild type and in *Wld^s^* mice, the number of infected oligodendrocytes should have been much lower in the latter, which was not the case. Therefore, the axon to myelin traffic of the virus took place in the absence of axonal degeneration in *Wld^s^* mutant mice.

Our results demonstrate that TMEV, a non-enveloped virus, is able to traffic from the axon into the surrounding myelin in the absence of membrane lysis. Non-lytic cell to cell transfer of a non-enveloped virus has remained a controversial point until now. In the case of persistent infections, non-lytic egress could be crucial by ensuring the survival of the infected host cell. Picornaviruses, such as TMEV, reorganize intra-cytoplasmic membranes to their advantage using pathways derived from the cellular process of autophagy. It has been proposed that the virus could egress from such infected cells in membrane bound vesicles derived from autophagosomes thereby escaping detection by neutralizing antibodies [8]. Viruses are masters at using cellular mechanisms for their own purpose and studying host-pathogen interactions is a very efficient way to uncover new functions in the host. It is most likely that TMEV is transferred from the axon to the myelin sheath through a mechanism that operates in normal, uninfected CNS. An exchange of cytoplasm between axon and myelin could be part of the support function of myelin for the axon. This would be consistent with the hypothesis, made on rather limited evidence, that myelin of the peripheral nervous system clears the axon from unwanted material [9] and on the results of ultrastructural studies suggesting the existence of cytoplasmic exchanges between axon and myelin [9]–[12]. Our paper may show the first instance where such an exchange is seen at work.

## Materials and Methods

### Mice

C57BL/6OlaHsd-Wld, referred to in this paper as *Wld^s^* mice, and their C57BL/6JolaHsd controls were purchased from Harlan, UK. All mice used were 4 week-old females.

### Experimental conditions to monitor axon-myelin traffic

Virus preparation, intraocular inoculation, quantification of viral load in the retina, preparation of optic nerve frozen sections, staining of the sections with fluorescent antibodies and microscopic analysis of the sections were described in detail previously [4].

### Analysis of the infection in the LGN

To measure infection in the LGN, 4% paraformaldehyde (PFA) fixed brains obtained as described previously [4] were infiltrated with 30% sucrose. Serial sections of the entire thalamus were prepared using a Leica sliding microtome. Free floating sections were stained for viral antigens using a 1/1000 dilution of an anti-TMEV capsid hyperimmune rabbit serum and an Alexa 555-labeled secondary anti-rabbit antibody.

### Detection of unphosphorylated neurofilaments with the SMI32 antibody

Anesthetized mice were perfused with PBS followed by buffered neutral formaldehyde (BNF) (33 mM NaH_2_PO_4_, 45 mM K_2_HPO_4_, 4% PFA). Mice were left intact for 1 hour. Optic nerves were then dissected out, post-fixed in BNF overnight at 4°C, and embedded in paraffin. Ten µm sections were cut with a microtome. Slides were baked at 56°C for 2 days, then deparaffinized and incubated for 40 minutes at 96°C in 10 mM sodium citrate buffer (pH6). They were then washed in water, blocked for 1h in PBS, 10% fetal calf serum (FCS) and 0.5% bovine serum albumin (BSA). The sections were incubated overnight at 4°C with the SMI32 antibody (Sternberger Monoclonals) diluted 1∶200 in PBS, 10% FCS, 0.5% BSA. After washing, the sections were incubated for 1 hour with an Alexa 488 coupled anti-mouse antibody diluted 1∶200 in PBS. Finally, the sections were mounted in Vectashield supplemented with DAPI (Vector). Sections were coded and observed blindly. All samples classified as showing axonal degeneration were from wild type mice upon decoding.

### Electron Microscopy

Samples were prepared for electron microscopy as described previously [13]. To assess axonal degeneration, EM grids of inoculated and control wild type and *Wld^s^* nerves were coded. Ten randomly selected regions imaged at x2.5k to x6.7k initial magnification were examined for change. All injected wild type and *Wld^s^* nerves were correctly identified as morphologically affected or normal, respectively.
